# Predicting single-cell cellular responses to perturbations using cycle consistency learning

**DOI:** 10.1093/bioinformatics/btae248

**Published:** 2024-06-28

**Authors:** Wei Huang, Hui Liu

**Affiliations:** College of Computer and Information Engineering, Nanjing Tech University, Nanjing, Jiangsu 211816, China; College of Computer and Information Engineering, Nanjing Tech University, Nanjing, Jiangsu 211816, China

## Abstract

**Summary:**

Phenotype-based drug screening emerges as a powerful approach for identifying compounds that actively interact with cells. Transcriptional and proteomic profiling of cell lines and individual cells provide insights into the cellular state alterations that occur at the molecular level in response to external perturbations, such as drugs or genetic manipulations. In this paper, we propose cycleCDR, a novel deep learning framework to predict cellular response to external perturbations. We leverage the autoencoder to map the unperturbed cellular states to a latent space, in which we postulate the effects of drug perturbations on cellular states follow a linear additive model. Next, we introduce the cycle consistency constraints to ensure that unperturbed cellular state subjected to drug perturbation in the latent space would produces the perturbed cellular state through the decoder. Conversely, removal of perturbations from the perturbed cellular states can restore the unperturbed cellular state. The cycle consistency constraints and linear modeling in the latent space enable to learn transferable representations of external perturbations, so that our model can generalize well to unseen drugs during training stage. We validate our model on four different types of datasets, including bulk transcriptional responses, bulk proteomic responses, and single-cell transcriptional responses to drug/gene perturbations. The experimental results demonstrate that our model consistently outperforms existing state-of-the-art methods, indicating our method is highly versatile and applicable to a wide range of scenarios.

**Availability and implementation:**

The source code is available at: https://github.com/hliulab/cycleCDR.

## 1 Introduction

Notwithstanding target-based drug discovery makes substantial advancements in recent years, intervention of a specific target (such as protein or RNA) by compounds or genetic perturbations does not consistently established systemic correlations with therapeutic outcomes or adverse effects at the organism level. Consequently, the failure rate of leading compounds generated from target-based screening to approved drugs remains high. As such, there has been an increasing interest in phenotypic drug discovery as a means to identify cell-active compounds.

Transcriptional profiling often serves as a molecular-level phenotypic measure of cellular responses to external perturbations. Large-scale compendia have been established to examine the perturbation-induced phenotypic alterations across a spectrum of cancer cell lines, including large-scale pharmacologic perturbation studies and cell viability measurements upon different drug treatments and genetic perturbations (Yang *et al.* 2012, Subramanian *et al.* 2017, Replogle *et al.* 2022). For example, the L1000 platform (Subramanian *et al.* 2017) is developed for high-throughput profiling of transcriptional responses of cancer cell lines subjected to a wide range of drug perturbations. In addition, proteomic responses to an array of clinically relevant drugs have also been documented using the reverse-phase protein arrays (RPPAs) technique. For example, the cancer perturbed proteomics atlas (CPPA) project has profiled >200 clinically relevant proteins, which covers major therapeutic targets in cancer therapy (Zhao *et al.* 2020), to drug perturbations. Transcriptional and proteomic profiling reflects the multi-level regulatory relationship transitions triggered by external perturbations, providing sound measurements of cellular states that could significantly aid phenotype-based drug screening efforts.

Moreover, the single-cell RNA sequencing (scRNA-seq) offers an unprecedented opportunity to discern subtle alterations in gene expression at single-cell resolution. Such level of precision is crucial for dissecting the effects of drugs or genetic interference on cellular state, and distinguishing cell subpopulations resistant to the treatment. For example, sci-Plex (Srivatsan *et al.* 2020) uses nuclear hashing to quantify global transcriptional responses to a number of independent perturbations at single-cell resolution. Replogle *et al.* (2022) conducts genome-scale Perturb-seq assays, which integrate CRISPR-based genetic screening with single-cell RNA-sequencing to chart the transcriptional response to genetic perturbations. The single-cell profiling facilitates the exploration of phenotypic heterogeneity among individual cells. Nevertheless, the capacity of the high-throughput screening technology is still limited relative to the vast space encompassing all possible combinations of individual cells and different perturbations. Therefore, the development of computational model emerges as a pivotal avenue for predicting single-cell responses to diverse external perturbations.

Some computational methods develop to predict cellular responses to perturbations. Among them, mechanistic modeling is leveraged to predict cell viability or the abundance of specific proteins (Li *et al.* 2019, Ferreira *et al.* 2020). While these models are favorable for interpretability (Fröhlich *et al.* 2018), they typically require longitudinal data that is often unavailable in practice. Furthermore, most mechanistic models do not scale well to genome-wide or high-dimensional scRNA-seq data, making them less suitable for predicting high-dimensional responses. In recent years, some methods have been proposed for predicting cellular responses to drug perturbations (Lopez *et al.* 2020, Hetzel *et al.* 2021). These methods include kernelized Bayesian matrix factorization (Madhukar *et al.* 2019), matrix factorization with similarity regularization (Gao *et al.* 2021), deep variational autoencoder (Jia *et al.* 2021), and convolutional neural network (Zhang *et al.* 2022). Other methods include low embedding of multiple genes, prior knowledge fusion (Chawla *et al.* 2022), mutational signatures (Aissa *et al.* 2021, Robichaux *et al.* 2021), and transcriptional expression (Sharifi-Noghabi *et al.* 2021, He *et al.* 2022). Two recent methods, CPA (Lotfollahi *et al.* 2021) and chemCPA (Hetzel *et al.* 2022), integrate linear model and adversarial learning to predict single-cell cellular response.

However, existing methods run into difficulty in modeling single-cell drug responses, primarily due to the absence of paired samples in the single-cell scenario. RNA-sequencing, which is a destructive process, precludes the possibility of profiling the transcriptional states of the same cell before and after perturbation. In this paper, we borrow the concept of cycle consistency, which is firstly proposed in cycleGAN (Zhu *et al.* 2017) for unpaired image-to-image translation, to model the unpaired single-cell data. Specifically, we require that unperturbed cellular state subjected to drug perturbations in a latent space would produce the actual cellular responses through a decoder network. Conversely, removal of drug perturbation from the perturbed cellular states would restore the unperturbed cellular states. Meanwhile, we postulate that the effect of external perturbations on cellular states follows a linearly additive model within the latent space, as adopted by previous studies (Lotfollahi *et al.* 2021, Hetzel *et al.* 2022). As such, the cycle consistency constraints can be bidirectionally imposed to simulate the drug exposure and drug withdraw operations. The integration of cycle consistency constraints and linear modeling enables to learn informative and transferable drug perturbation representations, so that our model can predict cellular response to previously unseen drugs with a high degree of accuracy. We evaluate our model on four distinct types of datasets, including bulk transcriptional drug responses, bulk proteomic drug responses, as well as single-cell responses to drug and gene perturbations. The empirical results demonstrate that our model consistently outperforms state-of-the-art methods, highlighting its potential to advance drug repositioning efforts.

We think this work have at least three contributions as below:

- To the best of our knowledge, we are the first to introduce cycle consistency constraints into the modeling of unpaired single-cell transcriptional responses to external perturbations. This enables our model to learn highly expressive and transferable representations of drug/gene perturbations.
- We establish the conceptual model of cellular response from two complementary viewpoints. Specifically, our model is required to simultaneously predict the cell state transitions from unperturbed state (control) to perturbed state (treatment) and vice versa. Such bidirectional constraints enforce the encoders to capture the essential feature of drug perturbations to cellular state.
- We evaluate our proposed model not only on bulk transcriptional and proteomic drug responses, but also two distinct single-cell response datasets. The comprehensive experimental results demonstrate the superior performance of our model, highlighting its versatility and reliability in predicting cellular responses to external perturbations.

## 2 Materials and methods

### 2.1 Encoder–decoder architecture

**Figure 1.**
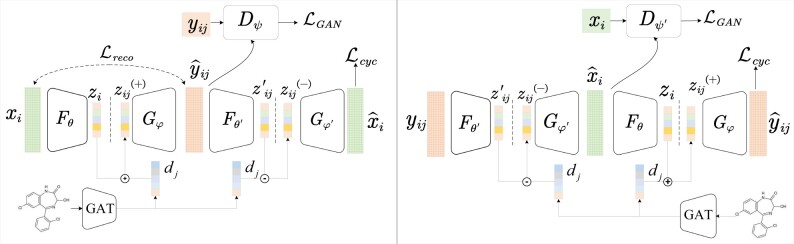
The illustrative diagram of cycleCDR learning framework. The model is based on autoencoder architecture, and two autoencoders learn collaboratively to mutually improve the ability of feature extraction for each other. The drug perturbation on cellular state is postulated to follow a linear additive model in the latent space, so that the cycle consistency constraints can be bidirectionally imposed to simulate the drug exposure and drug withdraw operations.

We use an encoder–decoder architecture to integrate the linear model and cycle-consistent constraints to predict cellular drug response. Figure 1 shows the illustrate diagram of our proposed learning framework. The molecular signatures stand for cellular state serve as the input of an encoder, which maps the cellular states of input samples into a latent space. The decoder endeavors to yield expected cellular states, depending on the embedding to be decoded is unperturbed or perturbed in the latent space.

Formally, we define the encoder as $F_{\theta }$, which maps each cellular state to an latent representation. The decoder is defined as $G_{\varphi }$ that converts the latent vector into output space. Denote by *x* and *y* the unperturbed and perturbed cellular states, and *z* represents the mapped latent representation whose dimension is equal to the size of autoencoder bottleneck layer. Our encoder/decoder networks are fully or densely connected neural networks with rectified linear unit (ReLU) activation function, θ and φ are the learnable parameters of the encoder and decoder. We require that the encoder–decoder architecture functions as an autoencoder so that the parameters are optimized to minimize the reconstruction loss as follows:

(1) $$L_{\mathrm{reco}}=\sum_{i=1}^{N}||x_{i}-G_{\varphi }(F_{\theta }(x_{i}))|{|}_{2}^{2}$$

The autoencoder converts the cellular states into low-dimensional but informative representations in the latent space. More importantly, in the latent space we build linear additive model for the cellular response to drug perturbation (see Section 2.3).

### 2.2 Drug encoder

The graph attention network (GAT) is used to encode the drugs into representations in the latent space. We use the SMILES descriptor of a drug to obtain its molecular graph *M*=(V,E), where *V* is the set of nodes (atoms) and *E* is the set of edges (chemical bonds). Assuming that $h_{k}$ is the embedding of node *k* and *W* is a learnable weight matrix, the attention score $\alpha_{kl}$ between node *k* and its first-order neighbor node *l* can be calculated using the following equation:

(2) $$\alpha_{kl}=\frac{\;\text{exp}(\mathrm{elu}(a^{T}(Wh_{k},Wh_{l})))}{\sum_{i\in N(k)}\;\text{exp}\;(\mathrm{elu}(a^{T}(Wh_{k},Wh_{i})))}$$

where *a* is a learnable vector, *elu* is the exponential linear unit activation function, and N(k) represents the first-order neighbor of node *k*. The attention score $\alpha_{kl}$ is actually the softmax normalized message between node *k* and its neighbors. Once the attention scores are computed, the embedding of node *k* is computed by aggregating its neighbor embeddings weighted by corresponding attention scores:

(3) $$h_{k}=\sigma (\alpha_{kk}Wh_{k}+\sum_{l\in N(k)}\alpha_{kl}Wh_{l})$$

where σ(.) is the ReLU activation function. Finally, we applied average pooling to aggregate the embeddings of all atoms to get the drug-level feature. For drug $d_{j}$, we obtained $d_{j}=\frac{1}{\left|V\right|}\sum_{n=1}^{\left|V\right|}h_{k}$, in which |*V*| represented the number of atoms of *j*th drug.

### 2.3 Linear modeling in latent space

Inspired by the linear additive model (Lotfollahi *et al.* 2021), we assume that the effect of drug perturbation on cellular states follows the linear additivity in the latent space. Given that the cellular state and drug disturbance are mapped to the same latent space, we construct a linear and easy-to-extend model. Assuming that the *j*th drug is mapped to $d_{j}$ via the drug encoder, the drug-induced cellular state in the latent space is defined as:

(4) $$z_{ij}^{\left(+\right)}=z_{i}+d_{j}$$

in which $z_{ij}^{\left(+\right)}$ represents the perturbed state of the *i*th cell by *j*th drug in the latent space. Assuming that the actual cell state induced by the drug is $y_{ij}$, the decoder $G_{\varphi }$ should yield ${\hat{y}}_{ij}=G_{\varphi }(z_{i}+d_{j})$ that approximates to actual response $y_{ij}$ as close as possible.

Meanwhile, based on the linear additivity assumption, the cellular response to drug perturbation can be modeled from the opposite direction. By subtracting the drug representation in the latent space, the drug effect should be withdrawn and the perturbed cellular state could be restored to the unperturbed state. Specifically, assume the encoder $F_{\theta^{{\;}^{\prime }}}$ maps the actual cellular response $y_{ij}$ to a embedding vector $z_{ij}^{\prime }=F_{\theta^{\prime }}(y_{ij})$ in the same latent space, withdraw of the drug perturbation can be modeled via subtraction of $y_{ij}$ by $h_{j}$. As a result, the drug-withdraw representation of the cellular state in the latent space can be defined as:

(5) $$z_{ij}^{\left(-\right)}=z_{ij}^{\prime }-d_{j}$$

where $z_{ij}^{\left(-\right)}$ represents the latent representation of the *i*th cell (single cell) after withdraw of the *j*th drug perturbation. Accordingly, another decoder $G_{\varphi^{\prime }}$ is used to map $z_{ij}^{\left(-\right)}$ back to the cellular state that should be as close as possible to $x_{i}$.

For scenarios with paired samples (unperturbed versus perturbed), we use the mean squared error loss function as below:

(6) $$\begin{array}{c} L_{\mathrm{MSE}}=\frac{1}{N*K}\sum_{i=1}^{N}\sum_{j=1}^{K}[{\left(y_{ij}-G_{\varphi }(z_{i}+d_{j})\right)}^{2}\\ +{\left(x_{i}-G_{\varphi^{\prime }}(z_{ij^{\prime }}-d_{j})\right)}^{2}] \end{array}$$

The first term corresponds to the loss in predicting the drug-perturbed cellular state from the initial state, while the second term corresponds to the loss in restoring the initial state from the drug-perturbed cell state.

However, in the context of single-cell transcriptional response to drug perturbation, the challenge arises from the loss of one-to-one correspondence of individual cells before and after drug exposure, due to the destructive nature of the single-cell RNA sequencing assay, which precludes direct tracking of cellular states across the perturbation. Therefore, we leverage adversarial learning to align the data distribution between two domains (unperturbed state versus perturbed state). In this situation, the decoder $G_{\varphi }$ functions as a generator that produces the perturbed cellular states, and a discriminator $D_{\psi }$ is introduced to distinguish between the actual and generated cellular responses. The adversarial loss is defined as follows:

(7) $$\begin{array}{c} \begin{array}{c} L_{\mathrm{GAN}}(G_{\varphi },D_{\psi },X,Y)=E_{\psi }[\text{log}\;D_{\psi }(y_{ij})]\\ +E_{\varphi ,\theta }[\text{log}(1-D_{\psi }(G_{\varphi }(z_{i}+d_{j})))] \end{array} \end{array}$$

where the generator $G_{\varphi }$ tries to produce cellular response $G_{\varphi }(z_{ij}^{\left(+\right)})$ that approaches to actual response as much as possible, while $D_{\psi }$ aims to distinguish between the generated and real ones. $G_{\varphi }$ aims to minimize the objective against an adversary $D_{\psi }$ that tries to maximize it, i.e. $\min_{G_{\varphi }}\max_{D_{\psi }}L_{\mathrm{GAN}}(G_{\varphi },D_{\psi },X,Y)$. Correspondingly, another discriminator $D_{\psi^{\prime }}$ is introduced to distinguish the actual unperturbed state from generated cell states by $G_{\varphi^{\prime }}$, and adversarial loss is defined as follows:

(8) $$\begin{array}{c} \begin{array}{c} L_{\mathrm{GAN}}(G_{\varphi^{\prime }},D_{\psi^{\prime }},Y,X)=E_{\psi^{\prime }}[\text{log}\;D_{\psi^{\prime }}(x_{i})]\\ +E_{\varphi^{\prime },\theta^{\prime }}[\text{log}(1-D_{\psi^{\prime }}(G_{\psi^{\prime }}(z_{ij}^{\prime }-d_{j}))] \end{array} \end{array}$$

### 2.4 Cycle consistency loss

Although adversarial learning-based domain adaption could align the data distribution of source domain and target domain, an encoder network has adequate capacity to map a specific set of cellular states to any random permutations in the latent space, which can lead to an output distribution that matches the target distribution well. Thus, adversarial loss alone cannot guarantee that the learned function can map an individual input to a desired output. To further reduce the space of possible mappings, we require that the learned mapping functions should be cycle-consistent.

Formally, suppose that the unperturbed cellular state $x_{i}$ is mapped to the drug-perturbed state ${\hat{y}}_{ij}$ by drug *j*, which should satisfy the requirement to restore the unperturbed state. We thus have $x_{i}\overset{G_{\varphi }(F_{\theta }(x_{i})+d_{j})}{\to }{\hat{y}}_{ij}\overset{G_{\varphi^{\prime }}(F_{\theta^{\prime }}({\hat{y}}_{ij})-d_{j})}{\to }{\hat{x}}_{i}\approx x_{i}$. From the opposite direction, the perturbed cellular state should be mapped to an unperturbed state that could be in turn used to restore its perturbed state, namely, we require $y_{ij}\overset{G_{\varphi^{\prime }}(F_{\theta^{\prime }}(y_{ij})-d_{j})}{\to }{\hat{x}}_{i}\overset{G_{\varphi }(F_{\theta }(\hat{x_{i}})+d_{j})}{\to }{\hat{y}}_{ij}\approx y_{ij}$. Thus, we define the cycle-consistency loss function as follows:

(9) $$L_{\mathrm{cyc}}=\sum_{i=1}^{N}{\left(x_{i}-{\hat{x}}_{i}\right)}^{2}+\sum_{i=1}^{N}\sum_{j=1}^{K}{\left(y_{ij}-{\hat{y}}_{ij}\right)}^{2}$$

### 2.5 Full objective

For bulk transcriptional or proteomic response data, we have the paired samples so that we define a relatively simple objective function:

(10) $$L=L_{\mathrm{reco}}+L_{\mathrm{MSE}}$$

For the single-cell cellular response to drug perturbation, we have no paired samples and thus define the full objective function as below:

(11) $$L=\alpha L_{\mathrm{reco}}+\beta L_{\mathrm{GAN}}+\lambda L_{\mathrm{cyc}}$$

in which $L_{\mathrm{GAN}}=L_{\mathrm{GAN}}(G_{\varphi },D_{\psi },X,Y)+L_{\mathrm{GAN}}(G_{\varphi^{\prime }},D_{\psi^{\prime }},Y,X)$, the hyperparameters (α, β and λ) are the weights standing for the importance of the three losses to the overall performance. In addition, similar to Taigman *et al.* (2016), we regularize the generator to produce nearly an identity mapping when real samples of the target domain (perturbed) are provided as the input to the generator. The identity loss is defined as $L_{\mathrm{identity}}=\sum_{i}^{N}\sum_{j}^{K}(G_{\varphi }(F_{\theta }(y))-y{)}^{2}$ and implicitly used in the full objective.

The two autoencoders defined in our model operate collaboratively to mutually promote their ability for feature extraction. We are also interested in using only one autoencoder for the cycle consistency leaning, namely $F_{\theta }=F_{\theta^{\prime }}$ and $G_{\varphi }=G_{\varphi^{\prime }}$. In fact, in our ablation experiment we confirm that dual autoencoder performs significantly better than single autoencoder.

## 3 Evaluation experiments

### 3.1 Experimental settings

The autoencoder and adversarial discriminator were realized using multi-layer MLPs with Relu activation function and batch normalization. The dimension of the bottleneck layer was set to 128. The drug molecular encoder consisted of two layers of graph attention networks (GATs) appended with a fully connected layer to adjust the embedding dimension. The parameters of the GAT encoder were initialized using the pre-trained model released by our prior work (Liu *et al.* 2022). The hyperparameter λ was set to 10 that was used by cycleGAN (Zhu *et al.* 2017).

To evaluate the performance of our model to predict cellular responses to drug perturbations, we computed the coefficient of determination ($r^{2}$) and explained variance (EV) between the actual and predicted on genome-wide expression profiles. To ensure reliable and objective performance evaluation, we randomly divided the dataset into training and test sets for model training and subsequent assessment. This process was independently repeated ten times to account for variations introduced by random data partitioning. Next, we calculated the standard deviation of each performance metric reported in the following experiments. In addition, because most genes remain almost unchanged upon external perturbation, the performance metrics computed over genome-wide expression profiles may not have accurately reflected the true predictive capacity of a model. Instead, a set of differentially expressed genes (DEGs) could more faithfully have reflected the actual effect of drug/gene perturbation on cellular state. Therefore, we also calculated the performance metrics based on the top 50 DEGs. The differential expression was defined as the logarithmic fold change >1 (${\;\text{log}\;}_{2}|fc|\geq 1$). On bulk datasets, the losses were evenly weighted. For single-cell datasets, the weight parameters α, β and λ were manually fine-tuned within the ranges of [1.5, 5], [1, 4], and [6, 7], respectively, to optimize performance for each individual dataset.

### 3.2 Performance evaluation on bulk transcriptional response

The L1000 dataset (Subramanian *et al.* 2017) was a collective repository of transcriptional responses of human cell lines to drug exposures. It consisted of ∼1 400 000 transcriptional responses of 50 cell lines to ∼20 000 drugs across a range of different concentrations. We obtained the data from L1000 website, and extracted the expression profiles of cell lines treated by 10 μM drug concentration. To streamline the data, technical replicates were averaged. As a result, we obtained drug-induced expression profiles spanning 6424 drugs and 42 cell lines. The refined dataset comprised a total of 45 763 expression profiles across 978 landmark genes. Since most cell lines exhibited weak response to drug perturbation, we focused our analysis on those cell lines that exhibited significantly differential expressions compared to their unperturbed states. Subsequently, the selected cell lines were randomly partitioned into validation and test set, while the remaining ones constituted the training set. As a result, we obtained the dataset with training (*n *=* *73 222), validation (*n *=* *9152), and test (*n *=* *9152) sets (for more detail see supplementary file).

**Figure 2.**
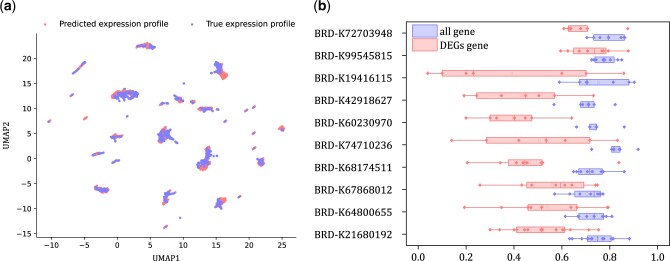
Performance evaluation on L1000 bulk transcriptional response data. (a) UMAP visualization of predicted and actual expression profiles of 2289 test samples. (b) The boxplots of $r^{2}$ scores regrading 10 drugs having the most number of perturbed cellular responses included in the test set.

To benchmark our model, we built a baseline model that calculated the performance metrics directly using the expression profiles of unperturbed and perturbed cellular state. Also, we compared our model with the chemCPA prediction model. As presented in Table 1, our model achieved the highest mean $r^{2}$ and EV scores among all comparative methods on the test set over all genes or the DEGs. The performance metrics of the comparative methods were sourced from their respective publications, which did not report confidence intervals. So, we have not provided standard deviations of these competing methods. These results strongly suggest that our model attained state-of-the-art performance. To provide a visual representations, we utilized the UMAP tool to visualize the actual and predicted gene expression profiles. As shown in Fig. 2a, the predicted values closely aligned with the actual values. Moreover, Fig. 2b presented the boxplots of $r^{2}$ scores for top 10 drugs that have been assayed on largest number of cell lines. Each point represented the predicted $r^{2}$ score regarding a cell line treated by one drug. It can be seen that our method achieved >0.75 mean $r^{2}$ scores across all genes, and 0.4–0.6 mean $r^{2}$ scores across DEGs. The experimental results indicated that our model effectively captured the effect of drug perturbations on gene expression.

**Table 1.** Performance comparison of cycleCDR with comparative methods on L1000 bulk transcriptional response dataset.

Model	Mean $r^{2}$ (All genes)	Mean EV (All genes)	Mean $r^{2}$ (DEGs)	Mean EV (DEGs)
Baseline	0.78 ± 0.02	0.78 ± 0.03	0.44 ± 0.02	0.60 ± 0.02
chemCPA	0.80	0.81	0.52	0.62
cycleCDR	**0.81** ± 0.02	**0.82** ± 0.03	**0.59** ± 0.02	**0.71** ± 0.03

The bold values represent the best performance.

### 3.3 Performance evaluation on proteomic response

Most molecular and targeted drugs mainly exerted their pharmacological effects by modulating protein function. The CPPA portal offered extensive proteomic expression data upon perturbation through RPPA assays (Zhao *et al.* 2020). The dataset reported the proteomic levels of 549 clinically relevant proteins across 126 human cell lines perturbed by 99 drugs. This rich resource enabled us to evaluate our model capacity in predicting the proteomic response to drug perturbation. After exclusion of the data points without dosage information, we obtained a refined dataset with 1760 drug-cell line combinations spanning 538 proteins. The processed dataset were randomly divided into training (*n *=* *2816), validation (*n *=* *352), and test (*n *=* *352) set (for more detail see supplementary file).

**Figure 3.**
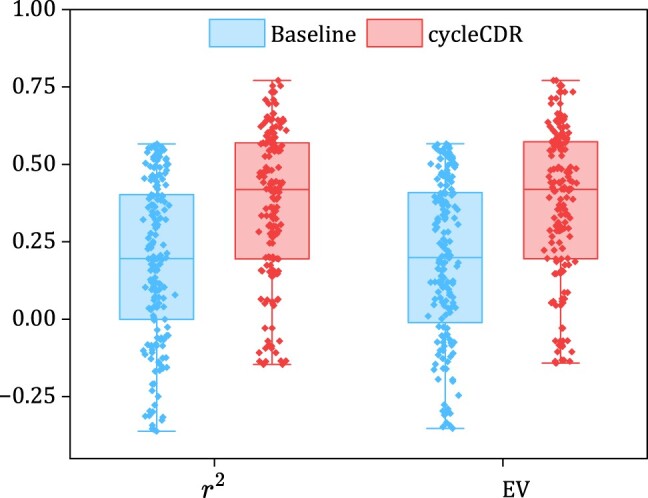
Performance evaluation of cycleCDR and baseline on CPPA bulk proteomic response dataset.

Because the CPPA dataset contained the proteomic profiles across varying drug dosages. To utilize this information, we transformed the drug dosage to an embedding and subsequently performed element-wise multiplication with the drug embedding itself. This allowed us to integrate dosage information into our predictive model. As a pioneer in predicting proteomic responses to drug perturbations, our performance comparison was limited to the baseline models. Figure 3 showed the mean and median $r^{2}$ and EV metrics on the test set. Compared to the baseline, our model showed notable superiority.

### 3.4 Performance evaluation on single-cell response to drug perturbation

The sci-Plex3 dataset (Srivatsan *et al.* 2020) provided the single-cell transcriptional responses of three human cancer cell lines (MCF7, K562, and A549) to 188 distinct drugs using high-throughput screening techniques. To ensure an unbiased comparison, we adopted the processed sci-Plex3 dataset by chemCPA (Hetzel *et al.* 2022) to evaluate our model predictive ability. The dataset were randomly divided into training (*n *=* *195 558), validation (*n *=* *14 634), and test (*n *=* *7894) sets (for more detail see supplementary file). We deliberately split the dataset into training and test set according to individual drugs. This ensured that all the data points associated with a specific drug exclusively appears in either the training or test set. The drug-level data partition strategy posed a challenge to check our model generalizability in predicting transcriptional response to unseen drugs during the training stage.

**Figure 4.**
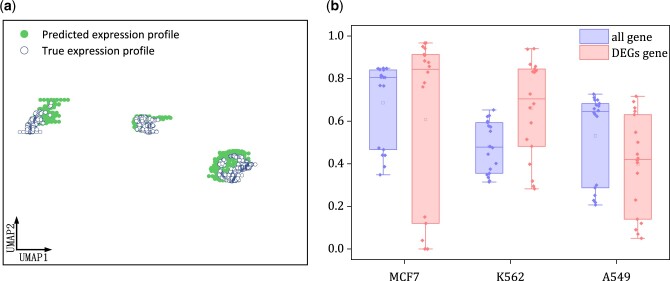
Performance evaluation on sci-plex3 single-cell transcriptional response dataset. (a) UMAP visualization of predicted and actual expression profiles. (b) Boxplots of $r^{2}$ values achieved by cycleCDR across all genes and DEGs on three cell lines.

For each cell treated by specific drug, we predicted its transcriptional profiles. Next, we computed the mean values of the expression levels for each genes across all the cells. As a result, we could compute the *r*^2^ score between the predicted and actual mean expression profiles. Table 2 presented the mean and median $r^{2}$ scores obtained on the held-out test set. Compared to chemCPA with and without pretraining on L1000 bulk data, our model significantly achieved better performance. This substantial improvement highlighted the effectiveness of our approach in predicting single-cell transcriptional drug responses. Furthermore, we used UMAP to visualize the predicted transcriptional profiles, and found they aligned closely to the actual ones, as shown in Fig. 4a. Figure 4b showed the boxplots of the $r^{2}$ scores across all genes and DEGs for each of the three cell lines (MCF7, K562, and A549) under drug perturbations. Each point in Fig. 4b represented the $r^{2}$ score corresponding to a drug-cell line pair. The experimental results showed that our method obtained ∼0.5 or more $r^{2}$ score for three cell lines, indicating that our model effectively predicted the single-cell transcriptional response to drug perturbations.

**Table 2.** Performance comparison of comparative methods on sci-Plex3 single-cell transcriptional response dataset.

Model	Mean $r^{2}$ (All genes)	Mean $r^{2}$ (DEGs)	Median $r^{2}$ (All genes)	Median $r^{2}$ (DEGs)
Baseline	0.37 ± 0.02	0.19 ± 0.02	0.16 ± 0.02	0 ± 0.02
chemCPA	0.46	0.22	0.35	0
chemCPA + pretraining	0.69	0.47	0.79	0.62
cycleCDR	**0.72** ± 0.03	**0.55** ± 0.03	**0.80** ± 0.04	**0.69** ± 0.03

The bold values represent the best performance.

We noticed that sci-Plex project had released an updated version, sci-Plex4, thereby went further to use this new dataset to evaluate our model (for detail of sci-Plex4 see supplementary file). Following similar data preprocessing and data partition strategy, the sci-Plex4 dataset was randomly divided into training (*n *=* *8140), validation (*n *=* *1436), and test (*n *=* *1466) sets (for more detail see supplementary file). We presented the mean and median ($r^{2}$) scores and explained variance (EV) on the held-out test set in Supplementary Fig. S1a and b. As expected, our model consistently outperformed the baseline model on all evaluation metrics. To further illustrate the effectiveness of our model, we presented an illustrative example focusing on the drug Quisinostat impact on individual K562 cells. Specifically, we selected the eight genes whose expression levels are most significantly altered by Quisinostat, and displayed in Supplementary Fig. S2 the predicted expression levels with the actual values upon drug exposure. The close agreement between the predicted and actual values provides strong evidence that our model accurately captured the complex patterns of single-cell transcriptional responses to drug perturbations. We have also examined the performance in predicting the transcriptional response of individual genes across samples on sci-plex4 dataset. The results were represented using scatterplots and boxplots in Supplementary Fig. S3, which verified that our method significantly outperforms the baseline model. Finally, we checked whether the sample similarity between training and test set influenced the prediction accuracy for different samples or not (Supplementary Fig. S4), and found that our model performed more better in predicting the induced response for the drugs whose pharmacological properties were seen during training stage (for more details see Supplementary Section S5 in Supplementary File).

### 3.5 Performance evaluation on single-cell response to genetic perturbation

We also evaluated our model performance on the cellular response datasets from two distinct genetic perturbation assays (Replogle *et al.* 2022). The datasets included single-cell RNA-sequencing readouts of the entire transcriptome of individual K562 and RPE-1 cells. Following the data preprocessing and data partition strategy mentioned above, the K562 dataset yielded the training, validation and test set with 111 392, 2294, 2264 samples. The RPE-1 dataset yielded the training, validation and test set with 126 024, 1828, 1756 samples, respectively. We utilized a one-layer fully connected network to substitute the GAT encoder for encoding genetic perturbation.

**Figure 5.**
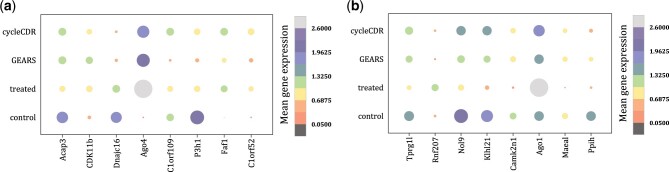
The actual and predicted expression levels for the eight genes whose expression are mostly altered in (a) K562 cell line induced by the SLU7 gene perturbation, and (b) RPE-1 cell line induced by SART1 gene perturbation.

**Figure 6.**
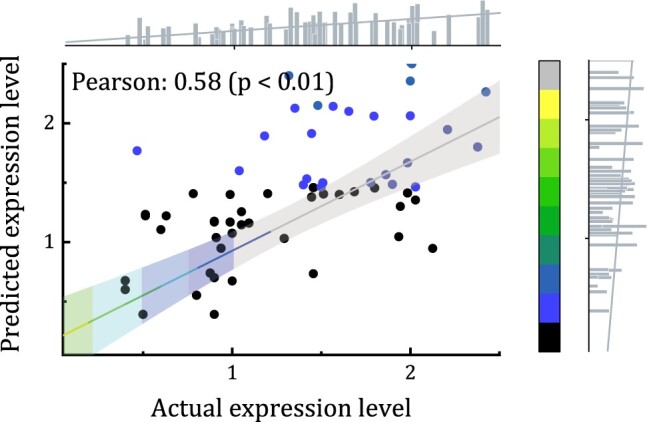
The scatter plot of actual and predicted transcriptional profiles of the three genes whose expression levels are mostly affected in the RPE-1 cell line with SART1 gene perturbed.

We conducted a comparative analysis with GEARS (Roohani *et al.* 2023), which integrated deep learning and knowledge graph of gene-gene relationships to predict single-cell transcriptional responses to genetic perturbations. The mean $r^{2}$ and EV values on the held-out sets of the two datasets were shown in Table 3. Notably, our model demonstrated superior performance across all genes and DEGs compared to the baseline and GEARS. Especially, our method achieved more remarkable advantage on predicting highly variable genes. For example, Fig. 5a illustrates the eight genes whose expression were mostly affected by the SLU7 gene perturbation in the k562 cell line. Similarly, Fig. 5b shows top eight genes whose expression are predominantly influenced by SART1 gene perturbation on the RPE-1 cell line. We observed that the predicted post-perturbation gene expressions closely aligned with the actual values. Moreover, for three genes (KLHL21, AG01, and MANEAL) that exhibited the most differential expression in the RPE-1 cell line induced by SART1 gene perturbation, we observed a significant correlation between predicted and the actual values (*P*-value <0.01), as presented in Fig. 6. The findings confirm the generalizability of our model in predicting differentially expression profiles induced by gene perturbations.

**Table 3.** Performance evaluation on replogle_k562 single-cell transcriptional response data.

Dataset	Model	Mean $r^{2}$ (All genes)	Mean EV (All genes)	Mean $r^{2}$ (DEGs)	Mean EV (DEGs)
K562	Baseline	0.84± 0.03	0.84± 0.02	0.10± 0.02	0.19± 0.02
	GEARS	0.86	0.86	0.29	0.32
	cycleCDR	**0.88** ± 0.02	**0.88** ± 0.02	**0.37** ± 0.03	**0.40** ± 0.03
RPE-1	Baseline	0.72± 0.02	0.72± 0.02	0± 0.02	0.11± 0.02
	GEARS	0.80	0.81	0.27	0.30
	cycleCDR	**0.82** ± 0.02	**0.82** ± 0.02	**0.45** ± 0.03	**0.46** ± 0.03

The bold values represent the best performance.

### 3.6 Model ablation

To verify the contribution of the individual model components to the performance enhancement, we set about to conduct model ablation experiments. The experiments included the evaluation of single autoencoder versus dual autoencoder architecture, as well as the reconstruction loss and cycle consistency loss. Table 4 showed the results of model ablation experiments performed on the sci-plex3 dataset, and revealed several notable findings. First, dual autoencoders notably outperformed single autocoder, due to the increment of parameter size. To confirm this point, we examined the loss curves generated during the training stage and found that the dual autoencoders achieve lower losses compared to single autoencoder.

**Table 4.** Performance evaluation of ablated models on sci-plex3 dataset.

Number of AE	$L_{\mathrm{reco}}$	$L_{\mathrm{cyc}}$	$\bar{r^{2}}$ (all)	$\bar{r^{2}}$ (DEGs)
Single AE	✓	✓	0.67±0.02	0.54± 0.03
		✓	0.56±0.02	0.46±0.03
	✓		0.45±0.03	0.38± 0.04
Dual AE	✓	✓	0.72±0.03	0.55±0.03
		✓	0.64±0.02	0.49±0.03
	✓		0.48±0.02	0.38±0.03

We also examined the effects of reconstruction loss and cycle consistency loss on the model performance separately, and found both loss terms contributed significantly. As expected, the cycle consistency loss contributed greatly to improve the model, especially for the prediction for DEGs. For instance, when using dual AE, the cycle consistency constraint notably enhanced the $r^{2}$ score for both all genes and DEGs, raising them from 0.48 and 0.38 to 0.64 and 0.49, respectively. This substantial improvement underscored the important role of cycle consistency loss in boosting model performance. In addition, Table 4 also demonstrated the importance of the reconstruction loss of the autoencoder. When combined, our model achieved best performance, implying that these two losses complemented each other effectively.

## 4 Discussion and conclusion

The model in predicting cellular responses to novel perturbations (Yu and Welch 2022) holds significant promise for drug repurposing. This endeavor necessitates our understanding of the intricate interplay between chemical compounds and cellular molecules, which can trigger a cascade of biochemical reactions and ultimately influence the molecular phenotype. Although the biological processes involved in cellular response to drug perturbation are nonlinear in nature, we postulate the effect of the perturbations on cellular states follows a linearly additive model in a latent space. The nonlinearity is assumed to be automatically handled by the deep neural network. More importantly, this assumption allows for a simplified yet informative representation of the complex interactions. In fact, the linear additive model in latent space has been adopted in previous studies (Lotfollahi *et al.* 2021, Hetzel *et al.* 2022). For example, the kernelized Bayesian matrix factorization (Madhukar *et al.* 2019) and matrix factorization with similarity regularization (Gao *et al.* 2021) actually fall in the community of linear models in low-dimensional latent space. Although these models are easy-to-interpret, their accuracy is still insufficient for drug discovery. We make advantage of the linearly additive models and the power of deep learning to greatly improve the predictive performance. Moreover, by virtue of the linear additivity assumption in the latent space, our method can be easily extended to scenarios involving multiple drugs, Taking two drugs as a representative case, we just need to use two GAT encoders to map drugs into the latent space, and then linearly combine their perturbations.

It is indeed noteworthy that our work shares some commonalities with CPA (Lotfollahi *et al.* 2021) and chemCPA (Hetzel *et al.* 2022) in terms of the integration of linearly additive model and deep learning for modeling cellular responses to perturbations. However, our method differs from CPA and chemCPA in at least three aspects. First, we introduce GAT encoder, rather than RDKit-based feature engineering, to learn drug representations from chemical structures, fully making advantage of the power of graph neural network. This not only enhances the capacity of your model for drug feature extraction, but also helps to establish an end-to-end model. Second, we introduce cycle consistency to address the challenge of unpaired samples encountered in single-cell data. The cycle consistency enables our model to learn informative and transferable drug perturbation features within various cellular contexts. Last, we incorporate actual unperturbed samples into the model training, whereas CPA and ChemCPA do not. The inclusion of real control data allows our model to more accurately capture the mapping between the unperturbed and perturbed cellular state. The flexibility and adaptability bring our method significant performance advantage over CPA and chemCPA. In addition, our method also differs from cycleGAN (Zhu *et al.* 2017) by using dual autoencoder architecture and introducing the drug perturbations in the latent space to impose bidirectional effect on cellular states.

In summary, we propose a novel deep network model to learn cellular response to drug perturbations. We integrate the cycle consistency loss and linear model in latent space into an end-to-end learning framework, which enables our model to learn expressive and transferable drug representations. We evaluate the proposed model on both bulk and single-cell transcriptional responses, as well as a proteomic drug response dataset. The experimental results on these benchmark datasets demonstrate the superior performance of our model.

## Supplementary Material

btae248_Supplementary_Data

## Data Availability

The source codes and datasets used in this study are available at: https://github.com/hliulab/cycleCDR.
