# Supplementary material for: Predicting single-cell cellular responses to perturbations using cycle consistency learning
Source: Bioinformatics. 2024 Jun 28;40(Suppl 1):i462–70. doi: 10.1093/bioinformatics/btae248 (PMC11256949; doi:10.1093/bioinformatics/btae248)
Supplement: btae248_Supplementary_Data [file btae248_supplementary_data.pdf]

Supplementary file of cycleCDR

Wei Huang, Hui Liu*

March 22, 2024

S1 Bulk transcriptional response to drug

The whole L1000 dataset comprises a total of 91,526 samples, covering 6,424 drugs and 42 cell lines. We identified the cell lines exhibiting significant gene expression changes compared to unperturbed cell lines, and randomly partitioned these cell lines to validation and test sets in 1:1 ratio. The remaining samples consists of the training set. As a result, we obtained the training, validation and test set as below:

- Training set: there are 73,222 samples in total, including 6,309 drugs and 42 cell lines.
- Validation set: there are 9,152 samples, including 3,003 drugs and 37 cell lines. This set served as an intermediate evaluation tool during training, facilitating parameter adjustments and mitigating the risk of overfitting.
- Test set: there are 9,152 samples, including 2,954 drugs and 40 cell lines. This set was employed to assess the final performance of the trained model.

S2 Proteomic response to drug

The RPPA dataset include 3520 samples in total, covering 156 unique drugs and 363 cell lines. We identified the cell lines exhibiting significant protein level changes compared to unperturbed cell lines, and randomly partitioned these cell lines to validation and test sets in 1:1 ratio. The remaining samples consists of the training set. Finally, we obtained the training, validation and test set as below:

- The training set comprises 2816 samples involving 52 drugs and 121 cell lines.
- The validation set consists of 352 samples regarding 52 drugs and 121 cell lines.
- The test set includes 352 samples, spanning 52 drugs and 121 cell lines.

S3 Single-cell transcriptional response to drug

The sci-Plex3 dataset comprises 218,086 samples (drug-cell pairs), spanning 187 drugs and 3 cell lines. We followed a similar approach to chemCPA by allocating

all cells treated with 9 drugs exclusively to the test set, ensuring that there is no overlap of these drugs between the training and test sets. This allows us to evaluate the model's ability to generalize to novel drugs. We identified the cells exhibiting significant gene expression changes compared to unperturbed cells, and randomly partitioned these cells to validation and test sets in 1:1 ratio. The remaining samples consists of the training set. Following data partition, we obtained the training, validation and test set as below:

- The training set includes 195,558 samples , covering 178 drugs and all 3 cell lines.
- The validation set contains 14,634 samples, covering 178 drugs and all 3 cell lines.
- The test set consists of 7,894 samples, focuses exclusively on the 9 held-out drugs across all 3 cell lines.

The sci-Plex4 includes a total of 11,042 samples (drug-cell pairs), encompassing 17 unique drugs and 3 distinct cell lines. Similarly, we identified the cells exhibiting significant gene expression changes compared to unperturbed cells, and randomly partitioned these cells to validation and test sets in 1:1 ratio. The remaining samples consists of the training set. Following data partition, we obtained the training, validation and test set as below:

- The training set includes 8,140 samples , covering 12 drugs and all 3 cell lines.
- The validation set contains 1,436 samples, covering 5 drugs and all 3 cell lines.
- The test set consists of 1,466 samples, focuses exclusively on the 5 held-out drugs across all 3 cell lines.

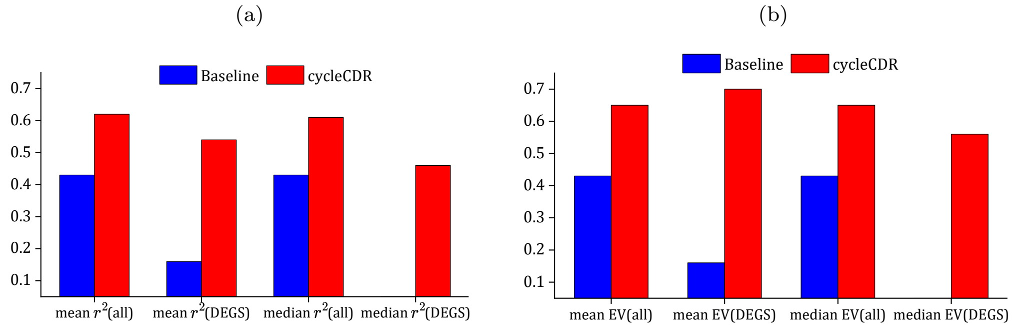

Figure S1: Performance evaluation on sci-plex4 single-cell transctiptional response dataset in the terms of r^2 and explained variance (EV) metrics.

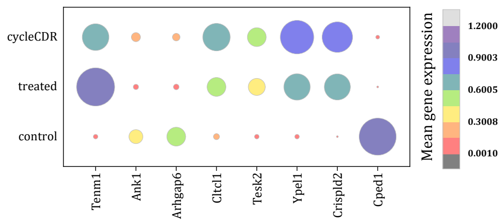

Figure S2: The actual and predicted expression levels of eight genes whose expression are mostly altered in K562 cell line induced drug Quisinostat assayed in sci-Plex4 dataset.

S4 Single-cell transcriptional response to gene perturbation

The Replogle et al. release the single-cell transcriptional responses to single-gene and multigene perturbations on K562 and RPE-1 cell lines. In our study, we consider only the single-gene perturbation data. Since most cells have limited response to gene perturbation, namely, most genes do not exhibit significant change in thier expression levels. To evaluate our model to capture the real response to gene perbutation, we identified the cells exhibiting significant gene expression changes compared to unperturbed cells, and randomly partitioned these cells to validation and test sets in 1:1 ratio. The remaining samples consists of the training set. The K562 dataset include a total of 116,050 samples (drug-cell pairs), encompassing 412 unique single-gene perturbation on one cell lines. Following data partition, the K562 dataset yield the training, validation and test set as below:

- The training set includes 111,392 samples, covering 397 gene perturbations on one cell lines.
- The validation set contains 2,294 samples, covering 15 gene perturbations and one cell lines.
- The test set consists of 2,264 samples, focuses exclusively on the 15 held-out gene perturbations on one cell lines.

The RPE-1 dataset include a total of 129,608 samples (drug-cell pairs), encompassing 651 unique single-gene perturbation on one cell lines. Following data partition, the K562 dataset yield the training, validation and test set as below:

- The training set includes 126,024 samples, covering 618 gene perturbations on one cell lines.
- The validation set contains 1,828 samples, covering 33 gene perturbations and one cell lines.
- The test set consists of 1,756 samples, focuses exclusively on the 33 held-out gene perturbations on one cell lines.

(a)

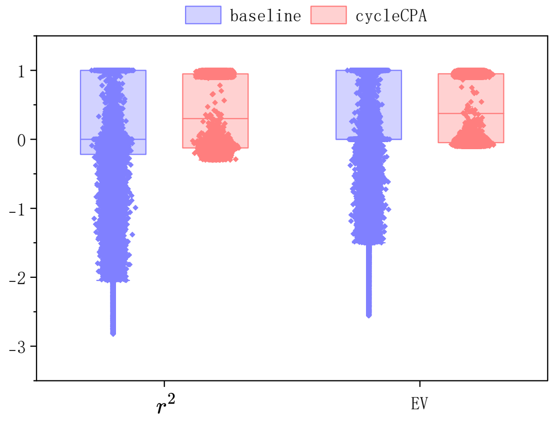

Figure S3: The boxplots of r2 scores computed for individual genes across samples on sci-plex4 dataset.

S5 Exploration of drug similarity influence on predictive performance

To explore the differences in prediction accuracy across different samples, we examined the similarity between training and test samples in the sci-plex4 dataset. Specifically, we considered each drug-cell line pair as a sample and calculated the similarity based on the induced gene expression profiles. We firstly computed the average values of the expression profiles across all cells of one type of cell line treated by a specific drug. The averaged profiles served as the representative for the drug-cell line sample. Subsequently, we calculated the cosine similarity. Figure 2a illustrates the similarity heatmap between the training samples (x-axis) and test samples (y-axis). Next, we employed the RDKit's Tanimoto algorithm to compute the chemical structure similarity between drugs. Figure 2b depicts the similarity heatmap for drugs in the training set (x-axis) versus those in the test set (y-axis).

Figure S4a clearly illustrated that the cell lines treated with Tucidinostat and Tacedinaline in the test set (denoted by green box) exhibited low similarity with the samples from the training set. In line with this observation, Figure S4b showed significant discrepancy in the chemical structures of these two drugs with those present in the training set (denoted by green box). The results imply a tendency for chemically similar drugs to induce similar gene expression profiles across the three cell lines studied.

We further check the model performance on these two drugs. Figure S4c presents the prediction results for the test set samples, with the x-axis representing cell line-drug samples and the y-axis denoting the r2 scores. Notably, the predictions for the samples associated with Tucidinostat and Tacedinaline are significantly lower than those for other samples.

Based on these observations, we hypothesize that the model is more accurate in predicting the effects of drugs with "seen" pharmacological properties during

training stage. This discovery underscores the importance of incorporating drug diversity to enhance the predictive accuracy. Such findings offers a new perspective on understanding model prediction biases and provides valuable guidance for future model improvements.

Table 1: Performance evaluation of sciplex4 single-cell transduction response data divided by cellular drug combinations.

Model	mean r^2 (All genes)	mean EV (All genes)	mean r^2 (DEGs)	mean EV (DEGs)
Baseline	0.44±0.02	0.44±0.02	0.25±0.02	0.25±0.02
cycleCDR	**0.59**±0.02	**0.64**±0.02	**0.49**±0.03	**0.63**±0.03

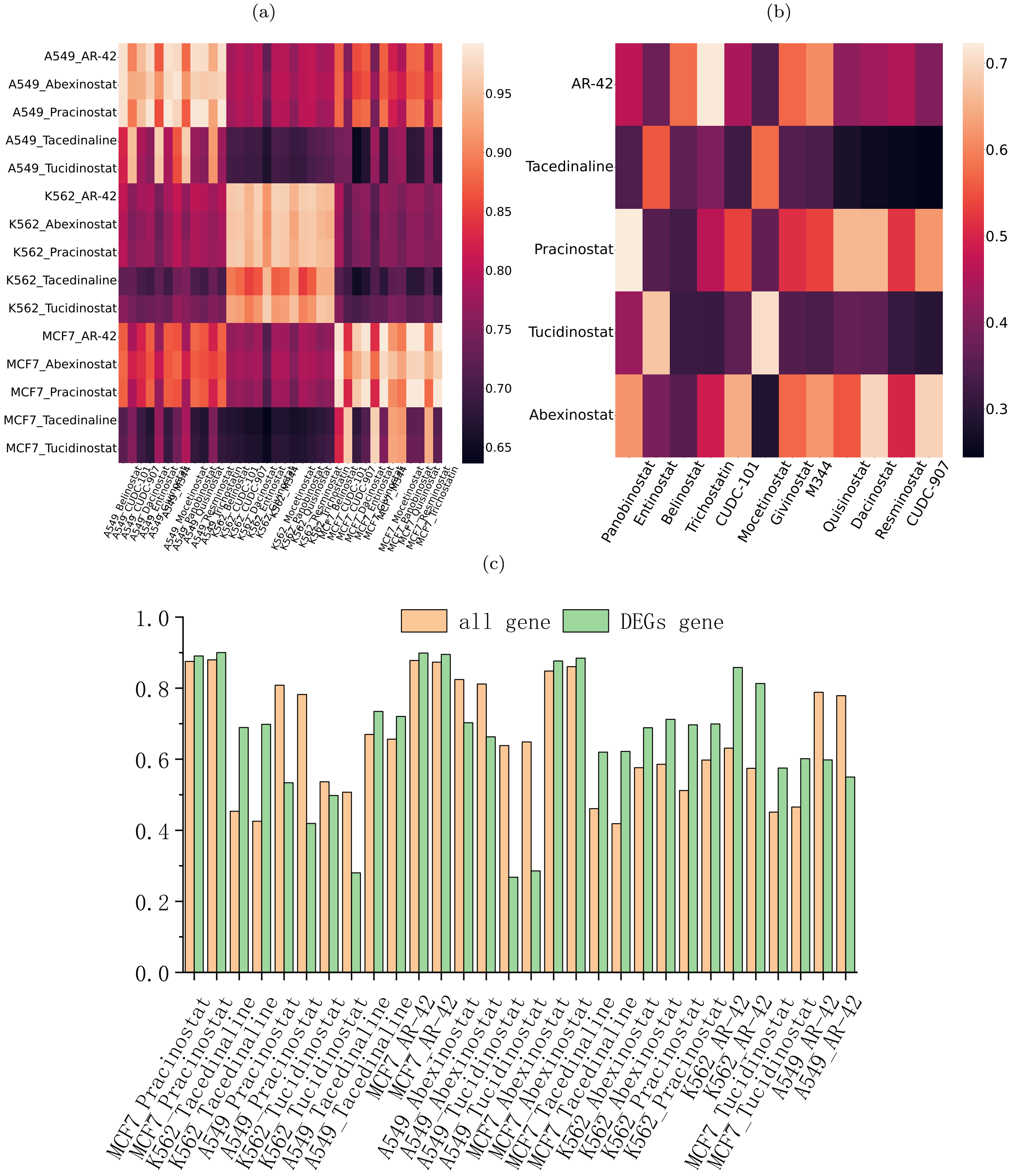

Figure S4: Performance evaluation of sciplex4 single-cell transduction response data divided by drug combination.(a) illustrated the similarity heatmap between the training samples (x-axis) and test samples (y-axis). (b) depicted the similarity heatmap for drugs in the training set (x-axis) versus those in the test set (y-axis).
